# Potential Plant–Plant Communication Induced by Infochemical Methyl Jasmonate in Sorghum (*Sorghum bicolor*)

**DOI:** 10.3390/plants10030485

**Published:** 2021-03-04

**Authors:** Felipe Yamashita, Angélica Lino Rodrigues, Tatiane Maria Rodrigues, Fernanda Helena Palermo, František Baluška, Luiz Fernando Rolim de Almeida

**Affiliations:** 1 Section of Plant Biology, Institute of Biosciences, São Paulo State University (UNESP), Botucatu 18618-689, Brazil; angelicarodrigues.bio@gmail.com (A.L.R.); tatiane.rodrigues@unesp.br (T.M.R.); fernanda.palermo@unesp.br (F.H.P.); luiz.rolim@unesp.br (L.F.R.d.A.); 2 Institute of Cellular and Molecular Botany, University of Bonn, 53115 Bonn, Germany; baluska@uni-bonn.de

**Keywords:** carbon assimilation, infochemical, plant signaling, photosynthesis, physiological memory, root anatomy, stomatal conductance

## Abstract

Despite the fact that they are sessile organisms, plants actively move their organs and also use these movements to manipulate the surrounding biotic and abiotic environments. Plants maintain communication with neighboring plants, herbivores, and predators through the emission of diverse chemical compounds by their shoots and roots. These infochemicals modify the environment occupied by plants. Moreover, some infochemicals may induce morphophysiological changes of neighboring plants. We have used methyl-jasmonate (MeJa), a plant natural infochemical, to trigger communication between emitters and receivers *Sorghum bicolor* plants. The split roots of two plants were allocated to three different pots, with the middle pot containing the roots of both plants. We scored low stomatal conductance (*g*_S_) and low CO_2_ net assimilation (*A*) using the plants that had contact with the infochemical for the first time. During the second contact, these parameters showed no significant differences, indicating a memory effect. We also observed that the plants that had direct leaf contact with MeJa transmitted sensory information through their roots to neighboring plants. This resulted in higher maximum fluorescence (*F*_M_) and structural changes in root anatomy. In conclusion, MeJa emerges as possible trigger for communication between neighboring sorghum plants, in response to the environmental challenges.

## 1. Introduction

The main cognitive functions of the nervous system, such as speech, memory, learning ability, and cognition, are strictly attributed to humans and some animals. Any attempt to compare such cognitive attributes to plants has been, and still is, labeled anthropomorphism, an attempt to humanize what is not human [1]. Obviously, plants have no neurons or a brain, so their sensory perceptions and the coordination of their organs must differ from those found in animals [2,3]. Nevertheless, plants are not senseless automatons and their adaptation and survival are based on plant-specific sensory systems continuously monitoring their environment [2,3,4,5,6,7,8].

Although plants are sessile organisms, they are able to actively move their organs (e.g., leaves and roots) and also to use these movements to interact with and manipulate the surrounding biotic and abiotic environments [4,5,6,7,8]. Plants generate electrical signals through membrane polarization and depolarization [9], as well as volatile chemical substances [6]. It is known that plants generate numerous different volatile substances, both from their shoots and roots [10], as well as root exudates [11]. These compounds help plants to communicate with herbivores, predators, and the parasites of their herbivores, and even with neighboring plants, may helping their defense strategy. However, for plant–plant communication to be accomplished, two factors are necessary. The first factor is that emitter plants exists. Second, it is necessary that receiver plants can capture, translate, and respond to these emitted signals [7]. The signal emitter can be a plant that, after an attack from a herbivore, activates mechanisms of response, triggering a cascade of internal signaling and long distance communication from the shoot to the root or vice versa. After this process of internal signaling, plant–plant signaling via volatiles can occur [12]. 

A relevant infochemical in plant signaling is methyl jasmonate (MeJa) [13]. This chemical compound is a phytohormone that acts as a natural plant regulator and plays a key role in a physiological pattern, plant growth, and development [13,14,15]. MeJa modulates root and shoot growth, leaf growth and senescence, pollen maturation, and formation of secondary metabolites [16,17]. This infochemical can also induce stomatal closure, consequently modifying water loss and CO_2_ absorption by the leaf, leading to a direct impact on photosynthetic machinery due to limited CO_2_ availability [17,18]. In addition, the exogenous application of MeJa can activate a signaling cascade for jasmonate production [19], inducing the accumulation of reactive oxygen species (ROS), inhibiting synthesis, and promoting the degradation of chlorophyll and rubisco, thereby causing a reduction in photochemical efficiency [20,21]. Consequently, plant growth and development can be modified.

Otherwise known as a stress hormone, MeJa and jasmonates plays a crucially role in response to biotic and abiotic stresses. In response to environmental stimuli, such as herbivory, plants typically release MeJa [13,15,22], and neighboring plants can capture this infochemical and begin a process of preparation and regulation of its defense mechanism against this biotic attack [14,23]. Recent research has shown that a slight touch of the aerial part from one plant to another can trigger responses in neighboring untouched plants through underground communication [24]. Still, in the same study, it was proven that roots have the ability to detect the altered physiological state of neighboring plants through chemical signals released as a root exudates. However, signaling to the environment through the roots [23,25,26] due to the contact of shoots with MeJa has not been examined thus far.

It is already known that after an initial stressful event, plants can modify their development patterns. In subsequent stressful events, plants can then adapt to environmental changes through a plant-specific learning process [27,28,29]. This learning process is based on developing anticipatory behavior without the need to learn from scratch during every environmental disturbance situation. Walter et al. (2013) called this learning process “stress memory” [30]. At the very beginning of a stressful event, the plant captures information (alarm phase) and throughout this period changes its physiological processes, which may promote the memory effect [30]. Therefore, these physiological adjustments can generate a stress “impression” that can enhance adaptive responses to subsequent stress events [28,29,30,31,32]. This process of memory in plants hardly resembles the neuronal networks and brains found in animals, but neurons may not be the only essential way of learning [33].

In this context, considering that plant communication can occur also through root–root signaling, the hypothesis of this study was that MeJa induces root communication between neighboring plants. The chemical signaling received by neighboring plants can cause morphophysiological alterations in receiver plants, which would favor tolerance to recurrent stress events. To test these hypotheses, this study aimed to evaluate: (i) The occurrence of changes in gas exchange and photosynthesis after first contact with MeJa; (ii) during the second contact, the plants become less sensitive to MeJa; (iii) neighboring plants can capture information about stressful events and alter their morphophysiological patterns accordingly. We designed a hydroponic experiment in which leaves of just one sorghum plant were exposed to MeJa, while their roots came into physical contact with the roots of neighboring plants using an experimental split root system [34].

## 2. Results

### 2.1. Assimilation Rate

We analyzed the effects of MeJa on the physiology of sorghum seedlings, applying the infochemical two times on the leaf surface of future emitter seedlings. 

**Figure 1:**
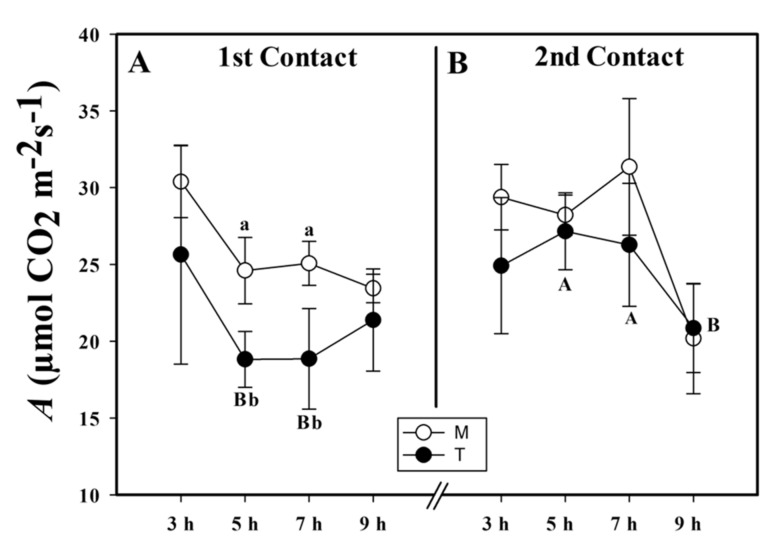
CO_2_ net assimilation (*A*, µmol CO_2_ m^−2^s^−1^) of the first (**A**) and second (**B**) contact with the methyl jasmonate (MeJa) (treated (T)) and mock (M) groups. MeJa was applied to the first fully expanded leaf of the plants in the T group at 6:00 a.m., 0 h after application (HAA), and evaluations were made at 3, 5, 7, and 9 HAA. The results are from a one-way ANOVA repeated measures, followed by Tukey’s test with a significance level of 5% (*p* < 0.05). The data refer to means (*n* = 4), error lines indicate standard deviation. Lowercase letters indicate differences between the T and M groups in the respective contact, while uppercase letters indicate differences between the first and second contact in the T group (*p* < 0.002).

In the first exposure to MeJa, we observed a smaller CO_2_ net assimilation rate (*A*) in the treated (T) plants in comparison to the mock (M) plants. In the T group, the *A* was smaller than that of the M group by 23.5% at 5 h and 20.4% at 7 h after application (HAA) (Figure 1A). Five days after the first contact, we applied MeJa for a second time and observed that *A* did not differ between the T and M groups (Figure 1B). In contrast, by just comparing the *A* of the plants that received the infochemical (T) between the first and second contact, we observed that the *A* was greater during the second contact than in the first contact by 44.31% at 5 and 31.67% at 7 HAA.

### 2.2. Stomatal Conductance

**Figure 2:**
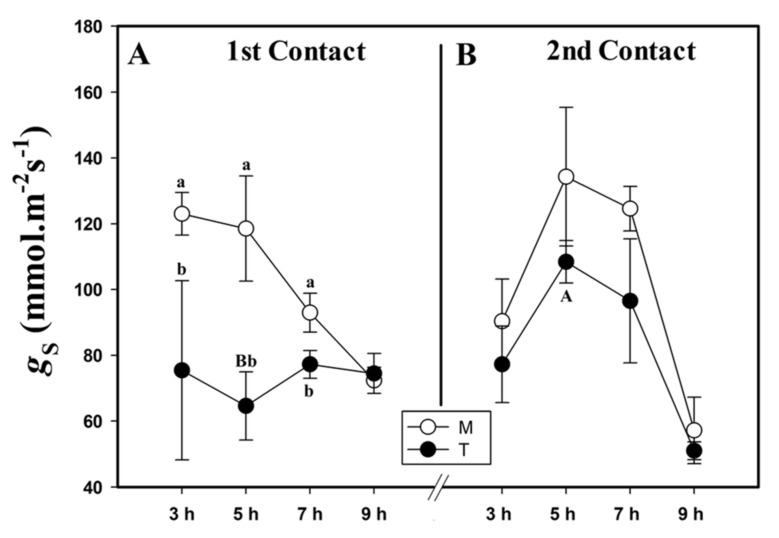
Stomatal conductance (*g*_S_, mmol.m^−2^s^−1^) of the first (**A**) and second (**B**) contact with MeJa (treated (T)) and mock (M) groups. MeJa was applied to the first fully expanded leaf of the plants of the T group at 6:00 am, 0 h after application (HAA), and evaluations were made at 3, 5, 7, and 9 HAA. The results are from a one-way ANOVA repeated measures, followed by Tukey’s test with a significance level of 5% (*p* < 0.05). The data refer to means (*n* = 4), error lines indicate standard deviation. Lowercase letters indicate differences between the T and M groups in the respective contact, while uppercase letters indicate differences between the first and second contact in the T group (*p* < 0.01).

Similarly to *A*, stomatal conductance (*g*_S_) decreased after MeJa contact. Just 3 HAA of MeJa, we observed smaller *g*_S_ in the plants of the T group compared to those of the M group. This pattern continued until at 7 HAA, only equaling out at 9 HAA (Figure 2A). We observed a 68% higher stomatal conductance of the plants in the T group during the second contact, when we compared it to the first contact at 5 HAA.

### 2.3. Maximum Fluorescence

**Figure 3:**
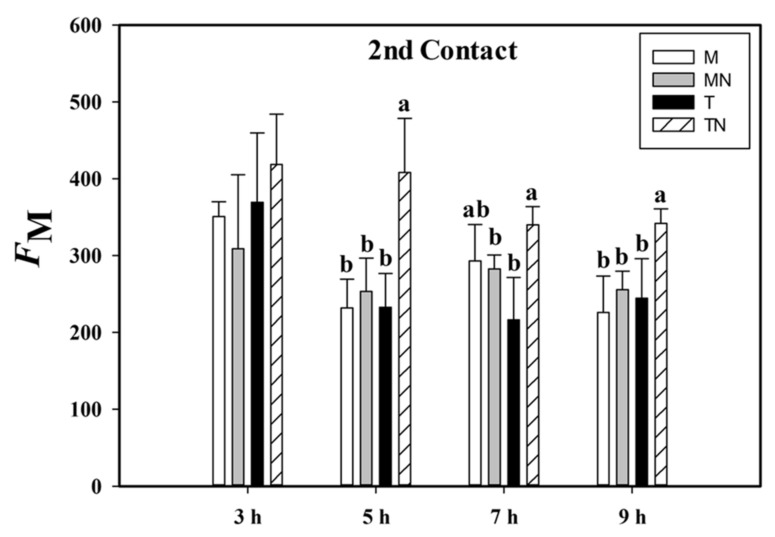
Maximum fluorescence (*F*_M_) of the second contact with the MeJa (treated (T)), mock (M), treated neighbor (TN), and mock neighbor (MN) groups. MeJa was applied to the first fully expanded leaf of the plants in the T group at 6:00 a.m., 0 h after application (HAA), and evaluations were made at 3, 5, 7, and 9 HAA. The results are from a one-way ANOVA repeated measures, followed by Tukey’s test with a significance level of 5% (*p* < 0.05). The data refer to means (*n* = 4), error lines indicate standard deviation. Lowercase letters indicate differences between the groups in the respective evaluations (*p* < 0.001).

The signaling led to changes in the physiological patterns of the stages in the photochemical phase of photosynthesis. During the second contact, we observed that the plants of the treated neighbor (TN) group had a higher maximum fluorescence adapted to light (*F*_M_) compared to the other groups. This difference was found in neighboring plants (mock neighbor (MN) × TN) at 5, 7, and 9 HAA during the second contact with MeJa. We also recorded the same difference in patterns at the same hours between the T and TN groups, being that the maximum fluorescence of TN was higher by 75.4% at 5 HAA, 57.3% at 7 HAA, and 39.9% at 9 HAA (Figure 3).

### 2.4. Anatomical Analyses of Adventitious Roots

The morphological analysis of the *Sorghum bicolor* adventitious roots after going through two rounds of contact with MeJa showed variations regarding the intercellular space in the cortex and the area occupied by the stele. Roots with smaller intercellular spaces were observed in the plants of the T and TN groups. The roots of the M group had a cortical intercellular space area that was 45.9% greater than that of the T group. The plants in the MN group had a cortical intercellular space that was 25.2% greater compared to that of the plants in the TN group. In contrast, the plants of the T and TN groups had larger steles.

**Figure 4:**
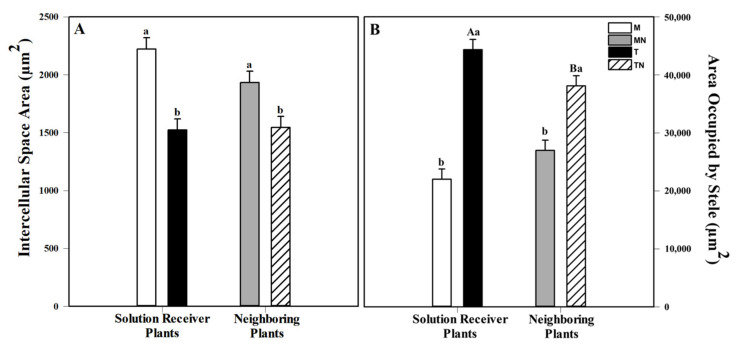
Anatomical aspects of *S. bicolor* on the 18th day of the experiment of all four groups: Mock (M), mock neighbor (MN), treated (T) and treated neighbor (TN). (**A**) Intercellular space area in the cortex (µm^2^). (**B**) Area occupied by the stele (µm^2^). The results are from a one-way ANOVA repeated measures, followed by Tukey’s test with a significance level of 5% (*p* < 0.05). The data refer to means (*n* = 4), error lines indicate standard deviation. Uppercase letters indicate differences between the plants that received the mock or MeJa solution and the neighboring plants (M × MN and T × TN), while lowercase letters indicate differences between the plants that received the solution and the neighboring plants (M × T and MN × TN) (*p* < 0.001).

The plant roots in the T group showed twice the area occupied by the stele (101.6%) in relation to those of the M group. The plants of the TN group showed roots with an area occupied by the stele that was 41.17% greater in relation to those of the MN group (Figure 4).

Discussed data that are not presented were collected, but no significant differences were observed. This data is in the Supplementary Tables S1 and S2.

## 3. Discussion

Contact of *S. bicolor* leaves with MeJa simulates stressful stimuli resembling herbivorous injuries and/or disease [22]. Only a few hours after contact with MeJa, we observed low stomatal conductance and carbon assimilation of the treated group (T) in relation to naive plants of mock group (M), especially the 5 h after application (HAA), the time of day when C4 plants are showing their optimal photosynthetic performance [35]. This might indicate that contact of the leaf with MeJa stimulated stomatal closure, reducing its conductance [21]. The strategy of stomatal closure triggered a series of physiological changes, one of them being the reduction of CO_2_ incorporation in the Calvin–Benson cycle and this lower carbon uptake reduced the net assimilation rate (*A*). In cases of photosynthesis reduction, this causes less sugar availability for the plant, consequently leading to the leaves consuming all of the sugar that they have already photosynthesized before complete stomatal closure. Depending on the severity and the stress prolongation, this can lead to senescence and eventual leaf fall, among other morphological responses [36].

During the second contact with MeJa, the plants of the T group had no variation regarding the mock group in the physiological parameters of either their stomatal conductance or their carbon assimilation, equalizing these parameters to those of the mock group. The MeJa-exposed plants may have demonstrated the ability to store information of the first contact and to react more quickly and efficiently in response to the second contact [37]. Initial exposure to stress can activate an epigenetic marker in a set of genes, facilitating faster and more efficient responses to future stresses [38]. This result may lead to the existence of physiological memory. This type of memory was named by Walter et al. (2011) as “stress imprint” [39], related to the phenotypic plasticity of a species. Regarding plant phenotypic plasticity, it is important to point out the plant specialization in a given environment; in other words, the greater the species plasticity, the greater is the acclimatization in contrasting environments [40].

The challenges induced by biotic and abiotic stress factors are interpreted by the plant after an internal signaling cascade has been accomplished [41], thus allowing the response of the whole plant. However, this signaling is not only restricted to the individual plant, but it can also be shared with the plants or organisms around them. This information can be shared by the roots, which are the main organ responsible for detecting the altered physiological state of their neighboring plants [24]. We tried to identify which chemical substance was responsible for the shared information between roots. For this, we analyzed the nutritive solutions of pots 1, 2, and 3 through HPLC–MS/MS. However, we did not find any differences in the solutions of the different pots, nor we did identify MeJa in the solutions (data not shown).

Although there were no differences in the nutritive solutions, we could observe the altered state in the maximum fluorescence (*F*_M_) patterns, indicating changes in photosystem II (PSII). This protein complex is among the first structures affected by exposure to stress [42]. Therefore, it is essential to re-organize the photosynthetic apparatus to dissipate the excess light energy absorbed in a metabolism weakened by a stressor. This regulation is observed with the chlorophyll *a* fluorescence parameter through photochemical and non-photochemical dissipation [43,44]. Our fluorescence data indicate that there may have been an indirect communication between plants, because chlorophyll fluorescence provides information about the PS II state [45] and damage to PSII reaction centers has been used to estimate the quantum efficiency of PSII [46]. Therefore, stressed plants with damaged photosynthetic tissues increase their nonphotochemical quenching processes, consequently decreasing *F*_M_ [47]. Nevertheless, our data show a higher *F*_M_ in the TN group. Even without any contact with MeJa, the plants of the TN group showed higher *F*_M_ in relation to the T and MN groups, increasing their fluorescence rates hours after the stimulus.

Physiological memory is indicated via the maximum observed fluorescence, when *F*_M_ could be an indicator of communication between adjacent plants, as well as the perception of stressors. In a previous study, similar effects were observed after sulfur dioxide exposure in an urban landscape [48]. This implies that damage promotes the rebuilding of photochemical apparatus and the optimization of physiological responses, bringing about better photochemical performance in recurrent stress via the stimulation of MeJa biosynthesis and signaling. Roots are well known to activate both jasmonate synthesis and signaling in response to shoot stress [49,50]. Intriguingly, even very weak mechanical stimuli induced by water droplets mimicking rain show this phenomenon [51].

Parallel to the physiological changes caused by the disturbance imposed on the plants, we observed structural changes in the roots of the plants in contact with MeJa and their neighbors. The roots from both groups of plants (T and TN) exhibited reduced area occupied by the intercellular spaces in the cortex and larger steles. Thus, concerning these anatomical parameters, it is remarkable that the naive neighbor plants responded to the MeJa treatment similarly to the treated plants, showing the structural plasticity of the tissues. In *S. bicolor*, the arrangement of the cortical cells is categorized as Panicoid-type and is characterized by the cuboidal packing of the inner cortical cells [52,53,54] with little extensive aerenchyma formation. The even smaller area occupied by the cortical intercellular spaces, as observed here in the roots of the plants of the T and TN groups, can be explained by a likely increased number of such cortical cells, an increased radial dimension of the parenchyma cells, or both. As it was observed in the roots of different plants under mechanical stress conditions [54,55,56,57], or can still be indirectly related to the higher stele size in these plants. The enlarged steles in the sorghum plants treated with MeJa (T) and in its neighbors (TN) could be associated with the overexpression of the genes related to stress, as reported for rice roots [58]. The stele size and the area occupied by the intercellular spaces in the cortex influence the rate of water and solute uptake by roots and their distribution between roots and shoots, involving coordinated activity of transport systems [59]. The structural parameters analyzed here could reflect important alterations in functioning of roots. Considering that the regulation of root water uptake is crucial to overcome stress injury [60], an increased volume of the stele may play an essential role in plant performance under these conditions.

## 4. Materials and Methods 

### 4.1. Plants and Growth Conditions

This study took place in a greenhouse under natural light conditions located in the Department of Biostatistics, Plant Biology, Parasitology and Zoology of the Institute of Biosciences of São Paulo State University (UNESP), Botucatu, Brazil. Gas exchange, chlorophyll *a* fluorescence, and morphostructural changes were evaluated.

*Sorghum bicolor* seeds of the BRS 332 variety were used. The seeds were provided by the Brazilian Agricultural Research Corporation (Embrapa Maize and Sorghum, Sete Lagoas, MG, Brazil) and were sown in styrofoam trays with vermiculite substrate and irrigated once a day for germination and rooting. 

At 20 days after sowing, the moment defined in this study as the juvenile growth phase, when the average height of seedlings was 25 cm, the seedlings were transplanted into a hydroponic system with Hoagland and Arnon (1950) nutrient solution nº 2, with 50% ionic strength, electrical conductivity of 1.2 mS, and pH 6.0 [61].

Plants were acclimatized for 10 days in different pots with a 500 mL capacity, filled with nutritive solution. This 10-day period was established after continuous physiological monitoring according to previous experiments of the research group [62] for better adaptation to the new culture medium and for the root growth needed to use specific techniques for this experiment.

During the whole experimental period, the condition of the greenhouse was monitored, with an average temperature of 27 °C, light intensity of 800 µmol m^−2^ s^−1^, and average relative humidity of 70%.

### 4.2. Plant–Plant Communication Experimental Design

**Figure 5:**
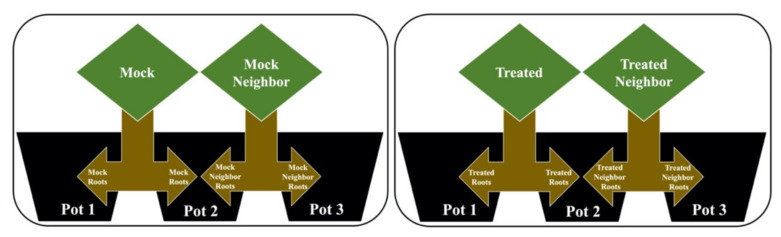
Experimental model for root communication following the model in [34,62]. The mock group had their roots divided and allocated to pots 1 and 2; the mock neighbor group had their roots divided and allocated to pots 2 and 3; the treated group had their roots divided and allocated to pots 1 and 2; the treated neighbor group had their roots divided and allocated to pots 2 and 3.

After the acclimatization period, the roots were split in two portions to assess the possibility of root communication induced by external stimuli. A randomized block design with four groups was used. The groups were as described below and shown in Figure 5:

(1) Mock (M): Contact with the mock solution (without the addition of MeJa), with its root separated into two parts, where half remained in pot 1 and the second half was allocated to pot 2.
(2) Mock neighbor (MN): Without contact with any solution, with its root also separated into two parts, where the first half was in pot 2, allowing direct contact with the roots of the mock group, while the second half was in pot 3.
(3) Treated (T): Contact with the MeJa solution, with its root separated into two parts, where half remained in pot 1 and the second half was allocated to pot 2.
(4) Treated neighbor (TN): Without contact with the MeJa solution, with its root also separated into two parts, where half was in pot 2, allowing direct contact with the roots of the treated group, while the second half was in pot 3.

In the proposed experimental model, designed according to Figure 5, pots with a capacity of 500 mL of nutritive solution were used (model adapted from [34,62]). The roots were separated in two parts, allowing physical contact between the roots of two different plants in the same pot (pot 2) and to verify the difference in exudates from the three different pots. Thirty days after germination (20 days in Styrofoam tray + 10 days for acclimatization in hydroponic system), treatment with MeJa (Sigma-Aldrich, Munich, Germany) was started to simulate a possible signal received by this chemical compound.

At 6:00 a.m. on days 1 and 10, we brushed 2 mL of the MeJa solution (in a Becker, 0.75 mM MeJa was diluted in 5% ethanol and 0.5% surfactant %*v/v*.) on the first fully expanded leaf until total exhaustion of the solution in the plants of the T group. The excess solution was gently removed with soft paper. For the M group, we used the same methodology, but with the mock solution (deionized water, ethanol, and surfactant). The solutions were not applied to the plants of the MN and TN groups. The surfactant agrex’oil (Microquimica Tradecorp, Campinas, SP, Brazil) was used to reduce the volatility and to enhance the adherence of the MeJa to the leaf surface, since the infochemical, despite being in the liquid phase, has volatile characteristics. For 4 days (96 h), the plants shared root exudates in pot 2, where there was contact between their roots. On day 5, all roots were carefully washed with deionized water for total removal of root exudates and the nutritive solutions of pots 1, 2, and 3 were replaced, starting the recovery period. This period lasted 5 days (days 5–10), until the physiological patterns returned to the initial state (same pattern as the day before the experiment started). On day 10, a new washing process took place, where a new nutritive solution was added to all of the pots and a new application of the MeJa and mock solutions occurred in their respective groups. On day 14, the nutritive solutions of all of the pots were replaced again, characterizing the end of the second cycle of the experiment. The end of the experiment occurred on day 18, with the collection of biological root material for anatomical analysis.

**Figure 6:**
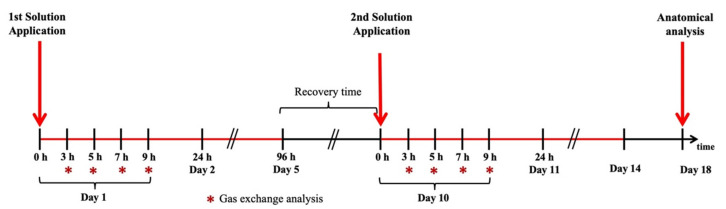
Time line of the experimental design for the methyl jasmonate (MeJa) application, gas exchange and anatomical analysis that were performed. The first and second red arrows show MeJa application at days 1 and 10 at 0 h. The third red arrow shows root collection for anatomical analysis. Red stars indicate gas exchange measurements.

Eight gas exchange analyses were performed to monitor the plants’ physiological state. Four analyses were performed on day 1 (first contact with MeJa) and the other four analyses were performed on day 10 (second contact with MeJa). Data collection of the gas exchange was performed in 4 repetitions per group and occurred at 3, 5, 7, and 9 h after application (HAA) of the MeJa or mock solution on the leaves, as shown in Figure 6.

The complete set of collected data is shown in the Supplementary Materials.

### 4.3. Physiological Analysis

Gas exchange measurements were carried out using equipment with an open photosynthesis system with CO_2_ and a water vapor analyzer by infrared radiation (Infra-Red Gas Analyzer (IRGA) and Fluorescence System, GFS 3000, Walz, Effeltrich, Germany). Analyses were carried out with four replicates per treatment at approximately 3, 5, 7, and 9 HAA (9:00 a.m., 11:00 a.m., 1:00 p.m., and 3:00 p.m., respectively) during the two application cycles, totaling 8 measurements. The evaluated parameters were the CO_2_ net assimilation (*A*, µmol CO_2_ m^−2^ s^−1^) and stomatal conductance (*g*_s_, mmol m^−2^ s^−1^). Measurements were standardized using the IRGA: 400 ppm for CO_2_ concentration, 20,000 ppm for H_2_O concentration, and 30 °C for leaf temperature.

The maximum fluorescence adapted to light (*F*_M_) was evaluated using a fluorometer (luminous intensity of 1500 µmol m^−2^ s^−1^ for photon flux density) coupled to the IRGA, so the times and number of evaluations were the same as those of the gas exchanges. Experiments were performed in the greenhouse with a constant average e temperature, air humidity, and vapor pressure deficit (VPD) (22.06 °C, 79.71%, and 0.54 kPa, respectively).

### 4.4. Morphological Analysis

Samples were taken from 0.5 cm above the tips of adventitious roots. The samples were fixed in FAA 50 (formaldehyde, acetic acid, 50% ethyl alcohol) [63] for 48 h and then stored in 70% alcohol. Afterward, they were dehydrated in ethanol series and embedded in methacrylate resin (Leica HistoResin, Leica, Wetzlar, Germany) [64]. The samples were sectioned on a semi-automatic rotary microtome and cross-sections (4-μm-thick) were stained with 0.05% Toluidine Blue pH 4.7 [65]. The slides were analyzed on a Leica DMR photomicroscope with DFC 425 camera (Leica, Wetzlar, Germany) attached. Quantitative analyses were performed using LAS software (V3.8 Leica, Wetzlar, Germany).

### 4.5. Data analysis

Data were analyzed using the statistical software SigmaPlot (12.0, Systat Software Inc. San Jose, CA, USA) All data were obtained from four biological repetitions and, after being submitted to the Shapiro–Wilk normality test (*p* < 0.05), were statistically analyzed by one way analysis of variance repeated measures (ANOVA). The mean values were compared by Tukey’s test (*p* < 0.05).

## 5. Conclusions

In *Sorghum bicolor*, during the first contact with MeJa, the plants of the treated (T) group showed changes in their physiological parameters. However, during the second contact, their responses did not differ from those of the mock (M) group, indicating that sorghum plants became less sensitive to MeJa after the first treatment. We also observed that the plants from the T group may have signaled their sensory information through their roots to their neighboring plants (i.e., the TN group). Nevertheless, our data do not exclude the contribution of shoot volatiles [66,67] in this plant–plant communication, since some studies have already demonstrated that it has an impact on gene expression and stomatal opening [68]. Altogether, MeJa may have led to plant–plant communication and altered the physiological and morphological patterns of the neighboring plants. In future, it will be important to study plant-plant communication from the perspective of critical physiological parameters of plant responses to environmental challenges, anticipating responses and increasing the chances of tolerating a possible future stress event. Intriguingly, in this respect, anesthesia induced with diethyl ether prevents both sensitivity to and accumulation of jasmonic acid in *Venus flytrap* plants [69]. Future studies should focus on the illumination of those mechanisms that interlink plant communication, behavior, and memory with jasmonate signaling related to the sensitivity of plants to anesthetics.

## Supplementary Materials

The following are available online at https://www.mdpi.com/2223-7747/10/3/485/s1, Table S1: Physiological analysis, Table S2: Anatomical analysis.

## Abbreviations

MeJa	Methyl jasmonate
ROS	Reactive oxygen species
*A*	CO_2_ net assimilation
T	Treated group
M	Mock group
HAA	Hours after application
g_S_	Stomatal conductance
TN	Treated neighbor group
MN	Mock neighbor group
*F*_M_	Maximum fluorescence adapted to light
VPD	Vapor pressure deficit
FAA 50	Formaldehyde, acetic acid, 50% ethyl alcohol
HPLC–MS/MS	High-performance liquid chromatography-mass spectrometry
