# Supplementary material for: Potential Plant–Plant Communication Induced by Infochemical Methyl Jasmonate in Sorghum (Sorghum bicolor)
Source: Plants (Basel). 2021 Mar 4;10(3):485. doi: 10.3390/plants10030485 (PMC8001897; doi:10.3390/plants10030485)
Supplement: Supplementary file 1 [file plants-10-00485-s001.pdf]

IZMBonn Institut für Zelluläre & Molekulare Botanik

UNIVERSITÄT BONN

Supplementary files

Table S1. Physiological analysis.

	HAA	Treatment	*A*	g_s	F_M
1st Contact	3h	Mock	30.39 ± 2.35	123.02 ± 6.46	347.75 ± 7.41
		Mock Neighbor	30.22 ± 1.13	84.04 ± 13.37	383.00 ± 26.61
		Treated	25.64 ± 7.13	75.44 ± 27.21	308.75 ± 72.49
		Treated Neighbor	26.90 ± 8.74	101.91 ± 47.58	301.00 ± 58.52
	5h	Mock	24.60 ± 2.16	118.52 ± 15.99	320.00 ± 67.05
		Mock Neighbor	28.62 ± 2.52	123.20 ± 7.89	334.00 ± 81.18
		Treated	18.82 ± 1.81	64.64 ± 10.35	213.25 ± 93.11
		Treated Neighbor	27.00 ± 8.27	97.22 ± 26.21	277.25 ± 79.28
	7h	Mock	25.08 ± 1.44	92.94 ± 5.89	258.25 ± 65.96
		Mock Neighbor	19.40 ± 1.96	70.12 ± 13.42	218.25 ± 20.02
		Treated	18.85 ± 3.28	77.25 ± 2.37	225.00 ± 84.06
		Treated Neighbor	23.76 ± 4.79	97.75 ± 17.76	252.50 ± 67.79
	9h	Mock	23.44 ± 0.92	72.37 ± 3.99	319.00 ± 84.03
		Mock Neighbor	22.29 ± 3.73	78.26 ± 5.73	340.75 ± 76.06
		Treated	21.37 ± 3.33	74.48 ± 6.09	291.50 ± 37.21
		Treated Neighbor	20.77 ± 8.21	73.20 ± 23.19	331.00 ± 47.38
2nd Contact	3h	Mock	29.39 ± 2.14	90.35 ± 12.86	351.00 ± 18.89
		Mock Neighbor	25.13 ± 1.77	87.97 ± 7.89	309.00 ± 96.09
		Treated	24.92 ± 4.44	77.27 ± 11.64	369.25 ± 90.25
		Treated Neighbor	26.66 ± 4.85	70.57 ± 20.29	419.00 ± 65.00
	5h	Mock	27.71 ± 0.82	134.28 ± 21.03	231.75 ± 37.77
		Mock Neighbor	29.70 ± 6.84	145.36 ± 28.27	253.50 ± 43.36
		Treated	27.16 ± 2.51	108.42 ± 6.49	232.75 ± 44.14
		Treated Neighbor	33.85 ± 1.66	138.68 ± 14.25	408.50 ± 70.32
	7h	Mock	31.36 ± 4.45	123.69 ± 6.75	293.25 ± 47.39
		Mock Neighbor	30.45 ± 2.40	105.72 ± 7.61	282.75 ± 18.28
		Treated	26.28 ± 4.01	94.92 ± 14.13	216.25 ± 55.49
		Treated Neighbor	30.78 ± 2.15	120.56 ± 5.72	340.25 ± 23.47
	9h	Mock	20.17 ± 3.59	57.17 ± 10.10	225.75 ± 47.67
		Mock Neighbor	22.96 ± 2.56	91.40 ± 6.74	255.50 ± 24.37
		Treated	20.84 ± 2.89	51.27 ± 2.83	244.25 ± 51.82
		Treated Neighbor	16.53 ± 2.73	48.49 ± 13.73	341.75 ± 18.98

Table S2. Anatomical analysis.

Groups	Treatment	Intercellular Space Area (μm²)	Area Occupied by Stele (μm²)
Solution Receiver	Mock	2221.14 ± 251,80	22,002.62 ± 1926.88
	Treated	1522.29 ± 149,89	44,356.55 ± 4975.44
Neighbor Plants	Mock Neighbor	1932.43 ± 175,90	26,987.43 ± 1421.13

	Treated Neighbor	1543.59 ± 186,90	38,100.53 ± 4357.96
